# Association Between the Waist–Triglyceride Index and Incident Type 2 Diabetes in the Elderly: A Retrospective Cohort Study

**DOI:** 10.1155/jdr/1555104

**Published:** 2025-11-20

**Authors:** Qianqian Wang, Zhaoxiang Wang, Yang Liu, Kaixin Zhou, Jinting Zhang, Qi Shao, Ming Kuang, Jia Tang, Ying Pan, Hongying Liu, Shao Zhong

**Affiliations:** ^1^Department of Endocrinology, The First People's Hospital of Kunshan, Kunshan, Jiangsu, China; ^2^Department of Critical Care Medicine, Shanghai East Hospital, Tongji University School of Medicine, Tongji University, Shanghai, China; ^3^Guangzhou Laboratory, Guangzhou, China; ^4^Kunshan Biomedical Big Data Innovation Application Laboratory, Suzhou, Jiangsu, China; ^5^Polifarma (Nanjing) Co. Ltd., Nanjing, Jiangsu, China; ^6^Department of General Medicine, The First People's Hospital of Kunshan, Kunshan, Jiangsu, China; ^7^Hangzhou Kang Ming Information Technology Co. Ltd., Hangzhou, Zhejiang, China

**Keywords:** cohort study, elderly, hypertriglyceridemia, Type 2 diabetes, waist circumference, waist–triglyceride index

## Abstract

**Introduction:**

Waist–triglyceride index (WTI) is a novel indicator of insulin resistance that can effectively identify glucose metabolism abnormalities and metabolic syndrome. No study discussed the predictive ability of WTI for incident Type 2 diabetes (T2D) risk from a longitudinal perspective. We aimed to evaluate the relationship between the baseline WTI level and the risk of T2D during follow-up in an elderly cohort.

**Methods:**

A total of 7578 participants aged over 60 years were enrolled in a follow-up study conducted from January 2018 to December 2023. The Cox proportional hazard models were performed to evaluate the independent effect of WTI level on T2D risk. The Kaplan–Meier method and restricted cubic spline (RCS) analysis were used to visually demonstrate the relationship between WTI and T2D risk. Receiver operating characteristic (ROC) curve analysis and decision curve analysis (DCA) were employed to evaluate the efficacy of WTI and other composite lipid parameters in assessing the risk of T2D.

**Results:**

During a median follow-up of 3.91 years (interquartile range: 2.89–4.86 years), 758 participants (10.0%) developed T2D. Fully adjusted Cox proportional hazard models demonstrated a positive and independent association between WTI and T2D risk (HR = 1.57, 95% CI: 1.37–1.80, *p* < 0.001). The highest WTI group (Q4) exhibited the greatest cumulative incidence of T2D (log-rank test, *p* < 0.001). Additionally, the RCS analysis indicated that the relationship between WTI and T2D risk was linear. ROC analysis and DCA suggested that WTI performed better in the diagnosis and prediction of T2D risk. Subgroup analysis further validated the stability of these findings.

**Conclusion:**

According to our study, the elderly with an elevated WTI level were at a higher risk of incident T2D. WTI may serve as a promising novel biomarker for T2D in large-scale epidemiological studies.

## 1. Introduction

Type 2 diabetes (T2D) is a common chronic metabolic disease [1]. In recent years, with improvements in living standards and changes in dietary structure, the incidence of T2D has been steadily increasing [2]. It was estimated that the global number of adults with diabetes reached 529 million (with over 90% being T2D), and this number is predicted to be 1.31 billion by 2050 [3]. Emerging as a major public health challenge worldwide, T2D and its concomitant complications impose significant health and economic burdens on patients and society. Notably, the incidence of T2D is higher in the elderly, and compared to younger patients, blood glucose management is more critical for elderly T2D patients because of increased risks of complications such as chronic kidney disease, cardiovascular disease (CVD), and cognitive impairment [4, 5]. Given the irreversible nature of T2D, early identification and management are particularly important [6]. Therefore, screening for simple and cost-effective predictors of T2D in high-risk elderly populations has become urgent.

Obesity is prevalent among individuals with T2D. Multiple studies have indicated that obesity, particularly visceral obesity, is closely associated with insulin resistance (IR) and an increased T2D risk [7, 8]. Traditional obesity indicators such as body mass index (BMI), waist circumference (WC), and waist-to-height ratio (WHtR) are commonly used due to their practicality [9]. However, these methods are limited in differentiating between subcutaneous and visceral fat [10]. Gold-standard methods for identifying fat distribution, such as magnetic resonance imaging and computed tomography scans, are expensive and complex and involve radiation exposure [11, 12]. High blood lipid levels have also been identified as an independent risk factor for T2D [13, 14]. Among the recently proposed indices is the waist–triglyceride index (WTI), which simultaneously considers WC and TG levels [15]. Subsequent studies have shown that WTI is a reliable clinical indicator of visceral fat accumulation compared to individual parameters [16, 17]. Previous studies have demonstrated that WTI can better reflect fat distribution and serve as a simple and useful alternative predictive indicator for IR [18]. Cross-sectional studies suggested that WTI was a risk factor for metabolic syndrome (MetS) [19, 20]. Reports on the relationship between WTI and the incidence of T2D in the elderly population are limited.

Therefore, this study utilized health data from the elderly population cohort to clarify the relationship between WTI and the incidence of T2D in Chinese adults, aimed at providing evidence for early prevention and identification of T2D, thus improving health outcomes.

## 2. Methods

### 2.1. Study Population and Design

This population-based cohort study utilized data linked to electronic health records (EHRs) from Kunshan's elderly cohort. Utilizing the healthcare information management system of the medical consortium, Kunshan's elderly cohort collected residents' health records, annual physical examination data, and chronic disease follow-up information. Personal information was anonymized and cleansed to ensure participants' privacy. Detailed cohort design information has been described previously [21–24].

**Figure 1:**
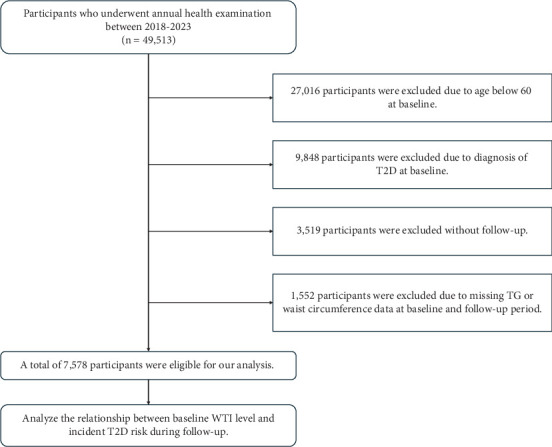
The flowchart of baseline population inclusion.

We included elderly residents who underwent community health check-ups in Kunshan between January 2018 and December 2023. Following ethical requirements to protect participant privacy, sensitive information such as names, identification numbers, and contact details was anonymized using a unified global index as the unique identifier for each participant. Participants were excluded if they met any of the following criteria: (i) aged less than 60 years at baseline, (ii) diagnosis of T2D at baseline, (iii) lack of follow-up information, or (iv) insufficient TG or WC data at baseline and follow-up period. Ultimately, a total of 7578 participants without diabetes were enrolled in this study (Figure 1). The protocol was approved by the First People's Hospital Ethics Committee of Kunshan (Grant No. 2023-03-014-H01-K01). All participants provided written informed consent for the use of their comprehensive EHR data.

### 2.2. Exposure and Outcome Definitions

The primary independent variable was WTI at baseline, calculated as Ln [TG (mg/dL) × WC (cm)/2] [20]. The composite lipid profiles were recorded, including the plasma atherosclerosis index (AIP), lipoprotein combine index (LCI), Castelli's Index I (CRI-I), and Castelli's Index II (CRI-II). The calculation formulas were as follows: AIP = log (TG/HDL‐C); LCI = (TC × TG × LDL‐C)/HDL‐C; CRI‐I = TC/HDL‐C; CRI‐II = LDL‐C/HDL‐C.

The primary endpoint indicator for this study was incident T2D at subsequent follow-ups, based on the following diagnostic criteria: the ICD-10 codes (E11–E14), FPG ≥ 7.0 mmol/L. [22] The interval between T2D onset and baseline assessment was reported as the timing of T2D. For participants without reported T2D during follow-up, we determined follow-up duration by the interval between the baseline assessment and their final survey date.

### 2.3. Covariate Definitions

We collected comprehensive clinical characteristics from the EHR health examination database, encompassing demographic information, annual lifestyle questionnaires (smoking status, drinking status), and anthropometric measurements (height, weight, WC, and blood pressure). Annual laboratory evaluation for the elderly included aspartate aminotransferase (AST), alanine transaminase (ALT), blood urea nitrogen (BUN), serum creatinine (Scr), serum uric acid (SUA), total cholesterol (TC), triglyceride (TG), and fasting plasma glucose (FPG) [25]. BMI was calculated by dividing weight by the square of height. Based on the Chinese BMI classification standards, physique categories are defined as follows: 18.5–23.9 kg/m^2^ (normal weight), 24.0–27.9 kg/m^2^ (overweight), and ≥ 28 kg/m^2^ (obese) [26]. The estimated glomerular filtration rate (eGFR) was calculated using the Chronic Kidney Disease Epidemiology Collaboration formula [27]. The diagnostic criteria for hypertension included ICD-10 codes (I10–I15), an average systolic blood pressure (SBP) ≥ 140 mmHg, and/or an average diastolic blood pressure (DBP) ≥90 mmHg [28]. The CVD diagnosis encompassed coronary artery disease (ICD-10 codes: I20–I25) and cerebrovascular diseases (ICD-10 codes: I60–I64), as documented in the EHR database [25]. The chronic disease registry and follow-up databases detailed the incidence, management, and outcomes of these conditions. Additionally, outpatient prescription and biochemical data improved the accuracy of T2D outcome definitions.

### 2.4. Statistical Analyses

The baseline characteristics of participants were presented as the mean ± standard deviation (SD) for continuous variables and as proportions (*n* [%]) for categorical variables. Differences among groups, based on the presence/absence of T2D or WTI quartiles, were assessed using either Student's *t*-test, ANOVA, or *χ*^2^ test. The Kaplan–Meier method was employed to assess the cumulative incidence of T2D events among WTI quartile groups, with differences measured using log-rank tests. Univariate and multivariate Cox analysis was performed to explore the associations between baseline WTI levels (as a continuous or categorical variable) and T2D risk. Clinically relevant and prognosis-associated variables were considered. Model 1 was unadjusted; Model 2 was adjusted for age and gender; Model 3 was further adjusted for BMI, FPG, ALT, AST, BUN, Scr, SUA, eGFR, TC, SBP, DBP, smoking status, drinking status, hypertension, and CVD. Using a restricted cubic spline (RCS) model with four knots, we also analyzed the dose–response association between baseline WTI and T2D risk. Receiver operating characteristic (ROC) curve analysis and decision curve analysis (DCA) were employed to evaluate the efficacy of WTI, WC, TG, AIP, LCI, CRI-I, and CRI-II in assessing the risk of T2D. Subgroup analyses were performed based on sex (male/female), age (≤ 70/> 70 years), BMI (normal weight/overweight/obese), CVD (yes/no), hypertension (yes/no), and eGFR (< 60/60–90/≥ 90 mL/min/1.73 m^2^) to validate the stability of the results. All statistical analyses were conducted using R Version 4.2.2 and Empower software (http://www.empowerstats.com). A two-sided significance level of *p* value < 0.05 was considered statistically significant.

## 3. Results

### 3.1. Baseline Characteristics

A total of 7578 participants without T2D at baseline were included in this study, with an average age of 66.93 ± 4.49 years, and 48.15% were male (Table 1). During a median follow-up of 3.91 years (interquartile range: 2.89–4.86 years), 758 (10.00%) participants developed T2D. Participants who developed T2D during follow-up were more likely to be older and female and have higher blood pressure, WC, BMI, ALT, AST, BUN, SUA, FPG, and TG levels, along with a higher prevalence of hypertension and CVD (*p* < 0.05). Conversely, eGFR was significantly lower in the T2D group (*p* < 0.001). Note that the WTI level of the T2D group was significantly higher than that of the non-T2D group (8.79 ± 0.58 vs. 8.55 ± 0.54, respectively; *p* < 0.001).

The baseline characteristics of participants, categorized by WTI quartiles (quartile [Q]1: 7.00–8.18; Q2: 8.18–8.53; Q3: 8.53–8.91; Q4: 8.91–11.50) are presented in Table 2. Participants in higher WTI quartiles generally exhibited higher WC, BMI, SBP, DBP, ALT, SUA, FPG, TC, and TG levels, as well as a higher prevalence of hypertension than those in the lower WTI quartiles (*p* < 0.05). Conversely, BUN and eGFR levels were lower in the higher WTI quartile group (*p* < 0.001). Additionally, significant differences were observed in smoking, alcohol consumption, and AST levels across the WTI quartiles (*p* < 0.001).

### 3.2. Relationship Between WTI and T2D Incidence

Cox proportional hazard models were used to analyze the independent effects of WTI on T2D incidence. Univariate Cox analysis revealed significant associations between the incidence of T2D and the following variables: age, BMI, FPG, ALT, AST, BUN, SUA, eGFR, LDL-c, HDL-c, SBP, gender, hypertension, CVD, and WTI (all *p* < 0.05). Furthermore, after adjusting for confounding factors identified in the univariate analysis, multivariate Cox analysis indicated that age, BMI, FPG, ALT, BUN, LDL-c, HDL-c, SBP, gender, hypertension, CVD, and WTI remained significantly correlated with the incidence of T2D (all *p* < 0.05) (Table 3).

Furthermore, based on existing literature and clinical experience, confounding factors covering demographics, lifestyle habits, and health indicators were selected for further sensitive analysis using the Cox proportional hazard model. The results indicated that the WTI was a significant risk factor in the unadjusted, partially adjusted, and fully adjusted models (all *p* < 0.001). In the fully adjusted model (Model 3), each 1-unit WTI increase was associated with a 57% higher risk of T2D incidence (hazard ratio [HR]: 1.57, 95% confidence interval [CI]: 1.37–1.80, *p* < 0.001). Converting WTI from a continuous variable to quartiles, the multivariate HRs for T2D incidence were 1.32 (95% CI: 1.02–1.70) for Q2, 1.61 (95% CI: 1.25–2.07) for Q3, and 2.02 (95% CI: 1.57–2.60) for Q4 compared to the lowest quartile (Q1) (all *p* < 0.05) (Table 4).

**Figure 2:**
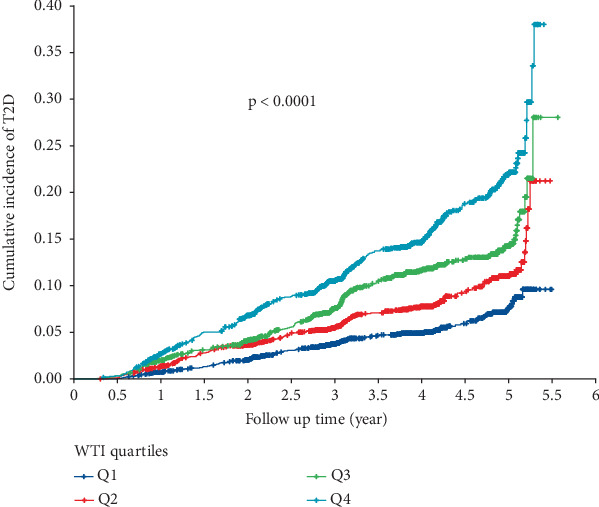
Kaplan–Meier curves according to quartiles of WTI (log-rank, *p* < 0.0001).

**Figure 3:**
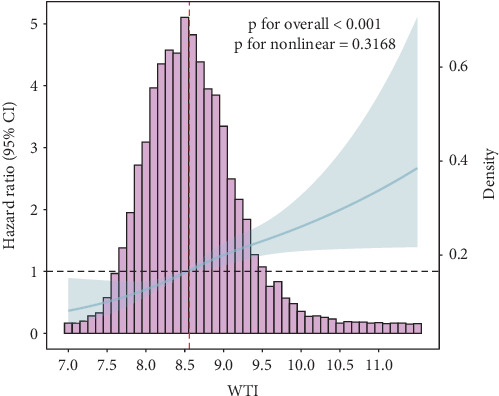
The association between WTI and incidence of T2D assessed by RCS analysis (adjusted for age, gender, smoking status, drinking status, hypertension, cardiovascular disease, BMI, SBP, DBP, FPG, ALT, AST, BUN, Scr, SUA, eGFR, TC).

As illustrated in Figure 2, the Kaplan–Meier curve showed that participants in the highest WTI quartile (Q4) had a significantly higher cumulative incidence of T2D during the follow-up period (log-rank test, *p* < 0.001). Furthermore, the RCS analysis demonstrated that T2D risk increased linearly with increasing WTI level (*p* for overall < 0.001 and *p* for nonlinear = 0.3168, respectively) (Figure 3).

**Figure 4:**
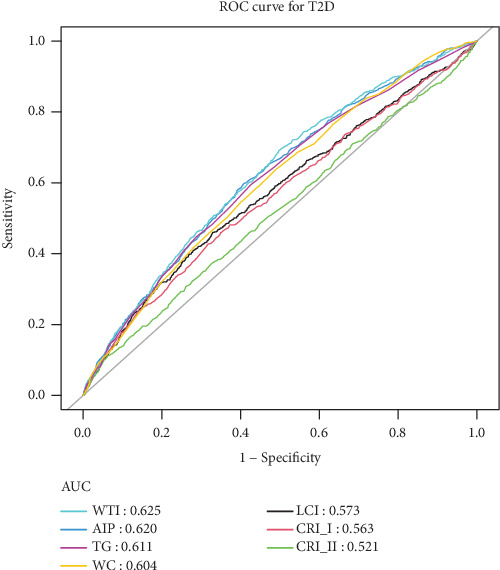
The results of ROC analysis. Abbreviations: WC, waist circumference; TG, triglyceride; WTI, waist–triglyceride index; AIP, plasma atherosclerosis index; LCI, lipoprotein combine index; CRI-I, Castelli's Index I; CRI-II, Castelli's Index II.

**Figure 5:**
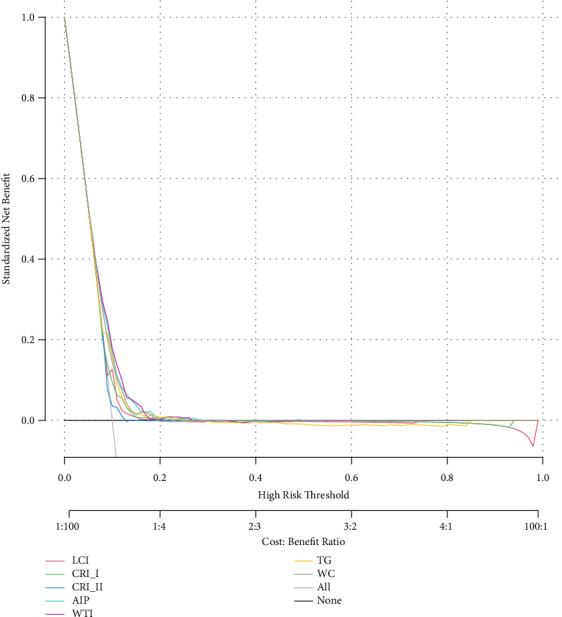
Decision curve analysis. Abbreviations: WC, waist circumference; TG, triglyceride; WTI, waist–triglyceride index; AIP, plasma atherosclerosis index; LCI, lipoprotein combine index; CRI-I, Castelli's Index I; CRI-II, Castelli's Index II.

Figure 4 presents ROC analysis results evaluating the WTI level and other composite lipid parameters for predicting the risk of incident T2D. Compared with TG, WC, AIP, LCI, CRI-I, and CRI-II, the WTI showed better performance with an area under the curve (AUC) of 62.5%. DCA also suggested that WTI performed better in the diagnosis and prediction of T2D risk (Figure 5).

### 3.3. Subgroup Analysis

**Figure 6:**
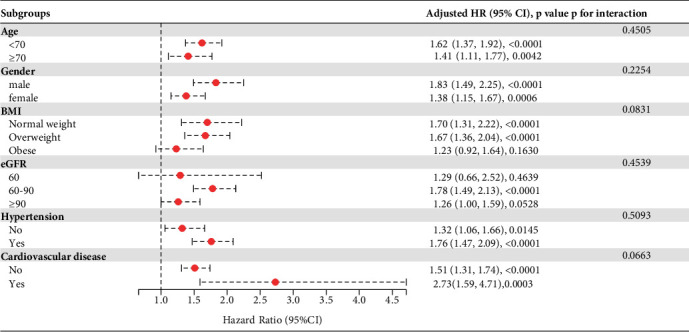
Subgroup analysis.

The association between the WTI and T2D risk was further analyzed in multiple subgroups stratified by sex (male/female), age (< 70/≥ 70 years), BMI (normal weight/overweight/obese), CVD (yes/no), hypertension (yes/no), and eGFR (< 60/60–90/≥ 90 mL/min/1.73 m^2^). As shown in Figure 6, the positive relationship between the WTI and T2D risk remained consistent across all subgroup variables (*p* for interactions > 0.05).

## 4. Discussion

In this large retrospective cohort study, we found that T2D occurs more frequently in participants with higher WTI levels at baseline. To our knowledge, we are the first to assess the relationship between WTI and T2D risk in the elderly cohort.

Multiple studies have validated the validity of obesity measures for predicting T2D risk [29]. Some scholars have found that even in people with normal BMI, central obesity (WHtR ≥ 0.5) is strongly associated with T2D [30]. Visceral fat may be a critical factor in differentiating metabolically healthy obese individuals from those who are not [31]. Due to the heterogeneity of obesity, the risk of metabolic abnormalities varies among individuals with different WCs, and TG levels were adopted as an additional indicator to measure visceral obesity better [32].

Multiple studies have underscored the critical importance of a comprehensive assessment of WC and TG levels for identifying individuals with elevated metabolic risk factors. The hypertriglyceridemic–waist (HTGW) phenotype, characterized by elevated WC and TG levels, has been demonstrated to be significantly associated with visceral fat accumulation and cardiovascular risk [33]. Several cohort studies have suggested that the HTGW phenotype constitutes a major metabolic risk for prediabetes and T2D [34, 35]. To further quantify the combined impact of TG and WC, Yang et al. introduced the waist circumference–triglyceride (WT) index, calculated as the product of WC and TG levels, and demonstrated a positive correlation between the WT index and the severity of the coronary lesion [15]. However, the utility of the WT index in predicting MetS remains contentious. Though the WT index exhibited superior predictive ability for high TG, blood pressure, and low HDL-C in women, it did not outperform WC alone in predicting MetS [36, 37]. Based on this, Liu et al. proposed a modified form of the WT index, termed WTI, as a novel IR surrogate index [20]. WTI was found to be a valuable parameter for early identification of IR, reflecting both abdominal fat distribution and lipid abnormalities. Subsequent studies have corroborated its utility in identifying MetS risk in nondiabetic and T2D adults [19, 38]. However, longitudinal evidence on the association between WTI and T2D risk is limited, particularly in high-risk elderly populations. In our study, we employed WTI to predict the incidence of T2D in elderly individuals, achieving consistent and promising results. These findings emphasize the clinical importance of assessing WTI in the elderly and may provide a rationale for better identifying subjects at high risk for diabetes. In addition, emerging comprehensive lipid indices, such as AIP, LCI, CRI-I, and CRI-II, have been shown to be associated with the risk of T2D [39, 40]. Our study suggests that WTI is a more effective predictor of T2D risk. However, it is important to note that while the AUC for WTI was 0.625, indicating statistical superiority over other tested indices, this value reflects only modest predictive accuracy in absolute terms. Therefore, while WTI serves as a valuable screening tool for risk stratification, its modest AUC suggests that it should not be used as a standalone diagnostic predictor. This nuanced understanding helps manage clinical expectations and underscores the need for a comprehensive approach to diabetes risk assessment.

Although the underlying mechanisms linking WTI to the pathogenesis of T2D remain unclear, several plausible explanations exist. Numerous epidemiologic and physiologic studies have noted that visceral obesity and dyslipidemia are central to the development of IR and aberrant glucose metabolism [41, 42]. Continued visceral fat accumulation contributes to adipocyte hypertrophy and increased macrophage infiltration, both of which secrete a range of adipocytokines and proinflammatory cytokines that promote, sustain, and exacerbate an insulin-resistant state [43]. In individuals with obesity, the dynamic equilibrium of lipid metabolism is disrupted, leading to excessive serum free fatty acids (FFAs), increased leptin, and decreased adiponectin levels, all of which can deteriorate insulin sensitivity and subsequently increase the risk of T2D [44]. Glucose homeostasis is closely related to lipid metabolism, with higher TG/HDL-c ratios, elevated TG levels, and lower HDL-c levels associated with an increased risk of developing T2D [45]. Concurrently, elevated WC and TG levels may indicate excessive lipid accumulation and consequent metabolic abnormalities [46]. As a marker of visceral obesity, elevated WTI levels are associated with an increased T2D risk, potentially mediated through IR mechanisms [47]. Further mechanistic research is necessary to elucidate the role of these indicators in the development of diabetes among elderly patients.

The strength of our study lies in its longitudinal evaluation of the association between WTI and the incidence of T2D, underscoring its significant practical value. Additionally, the study featured a long follow-up period, a low loss-to-follow-up rate, and a stable study population. Various methods for sensitivity analyses were employed, reinforcing the robustness of our findings.

However, there are potential limitations to this study. First, due to the secondary analysis nature of a retrospective cohort, some crucial variables, such as family history of DM and educational background, were unavailable. Family history is a potent, nonmodifiable risk factor for T2D. The inability to adjust for family history of diabetes represents a significant limitation of our study, as it may introduce residual confounding that could affect the observed association between the WTI and T2D risk. Consequently, this limitation may result in an overestimation of the reported HRs, underscoring the need for caution in interpreting our findings. Second, as the study only included elderly Chinese patients, generalizing the findings to other populations needs further investigation. Specifically, our cohort consisted of community-dwelling older adults, which may limit the applicability of our findings to other elderly populations, such as those residing in long-term care facilities who may have different health and nutritional profiles. Future research should include a more diverse range of elderly populations to enhance the generalizability of the findings and to explore how different living conditions may influence the relationship between WTI and T2D risk. Third, the relationship between WTI and diabetic complications warrants further exploration in subsequent follow-up studies.

## 5. Conclusion

In the elderly population, a high WTI level was associated with an elevated T2D risk during follow-up. Further studies are warranted to determine whether close monitoring or intervention to reduce WTI levels could improve health outcomes.

## Figures and Tables

**Table 1:** Baseline characteristics of the study population.

	**Total (** **N** = 7578**)**	**Nondiabetes group (** **N** = 6820**)**	**Diabetes group (** **N** = 758**)**	**p** ** value**
Age, years	66.93 ± 4.49	66.80 ± 4.38	68.06 ± 5.26	< 0.001
Sex, men	3649 (48.15%)	3329 (48.81%)	320 (42.22%)	< 0.001
WC, cm	84.77 ± 8.99	84.43 ± 8.95	87.80 ± 8.78	< 0.001
BMI, kg/m^2^	24.51 ± 3.23	24.36 ± 3.19	25.90 ± 3.28	< 0.001
Smoking				0.134
Never	5698 (75.99%)	5114 (75.82%)	584 (77.56%)	
Former	275 (3.67%)	257 (3.81%)	18 (2.39%)	
Current	1525 (20.34%)	1374 (20.37%)	151 (20.05%)	
Drinking				0.021
None	5969 (79.61%)	5337 (79.13%)	632 (83.93%)	
Light	490 (6.54%)	452 (6.70%)	38 (5.05%)	
Moderate	148 (1.97%)	137 (2.03%)	11 (1.46%)	
Heavy	891 (11.88%)	819 (12.14%)	72 (9.56%)	
SBP, mmHg	139.02 ± 19.17	138.62 ± 19.22	142.55 ± 18.32	< 0.001
DBP, mmHg	81.47 ± 10.65	81.37 ± 10.72	82.35 ± 9.94	0.016
ALT, U/L	19.84 ± 12.71	19.45 ± 12.47	23.34 ± 14.18	< 0.001
AST, U/L	22.49 ± 10.38	22.30 ± 10.08	24.18 ± 12.59	< 0.001
BUN, mmol/L	5.62 ± 1.49	5.60 ± 1.48	5.81 ± 1.58	< 0.001
Scr, *μ*mol/L	72.50 ± 21.46	72.41 ± 21.78	73.31 ± 18.28	0.272
SUA, mg/dL	324.97 ± 83.77	322.83 ± 83.13	344.34 ± 87.01	< 0.001
eGFR, mL/min/1.73 m^2^	84.52 ± 12.94	84.81 ± 12.76	81.88 ± 14.17	< 0.001
FPG, mmol/L	5.45 ± 0.66	5.39 ± 0.63	6.05 ± 0.57	< 0.001
TC, mg/dL	184.68 ± 35.78	184.80 ± 35.49	183.59 ± 38.29	0.374
TG, mg/dL	145.99 ± 107.66	142.24 ± 103.12	179.73 ± 137.70	< 0.001
Hypertension	3727 (49.18%)	3274 (48.01%)	453 (59.76%)	< 0.001
Cardiovascular disease	318 (4.20%)	235 (3.45%)	83 (10.95%)	< 0.001
WTI	8.58 ± 0.54	8.55 ± 0.54	8.79 ± 0.58	< 0.001

*Note:* Continuous variables are shown as mean ± SD, and categorical variables are presented as *n* (%) numbers.

Abbreviations: ALT, alanine transaminase; AST, aspartate transaminase; BMI, body mass index; BUN, blood urea nitrogen; DBP, diastolic blood pressure; eGFR, estimated glomerular filtration rate; FPG, fasting plasma glucose; SBP, systolic blood pressure; Scr, serum creatinine; SUA, serum uric acid; TC, total cholesterol; TG, triglyceride; WC, waist circumference; WTI, waist–triglyceride index.

**Table 2:** Baseline characteristics of the study population according to WTI quartiles.

	**Quartile 1** **(** **W** **T** **I** < 8.18**)**	**Quartile 2** **(**8.18 ≤ **WTI** < 8.53**)**	**Quartile 3** **(**8.53 ≤ **WTI** < 8.91**)**	**Quartile 4** **(** **W** **T** **I** ≥ 8.91**)**	**p** ** value**
*N*	1894	1894	1895	1895	
Age, years	66.91 ± 4.55	66.98 ± 4.55	66.94 ± 4.55	66.88 ± 4.30	0.921
Sex, men	1104 (58.29%)	958 (50.58%)	830 (43.80%)	757 (39.95%)	< 0.001
WC, cm	78.85 ± 8.29	83.88 ± 7.85	87.06 ± 8.06	89.26 ± 8.16	< 0.001
BMI, kg/m^2^	22.66 ± 3.07	24.11 ± 2.85	25.28 ± 2.98	26.00 ± 2.98	< 0.001
Smoking					< 0.001
Never	1328 (71.05%)	1429 (76.13%)	1479 (78.96%)	1462 (77.81%)	
Former	76 (4.07%)	64 (3.41%)	69 (3.68%)	66 (3.51%)	
Current	465 (24.88%)	384 (20.46%)	325 (17.35%)	351 (18.68%)	
Drinking					0.003
None	1420 (75.98%)	1493 (79.54%)	1525 (81.42%)	1531 (81.48%)	
Light	141 (7.54%)	126 (6.71%)	111 (5.93%)	112 (5.96%)	
Moderate	44 (2.35%)	39 (2.08%)	37 (1.98%)	28 (1.49%)	
Heavy	264 (14.13%)	219 (11.67%)	200 (10.68%)	208 (11.07%)	
SBP, mmHg	135.30 ± 19.16	137.60 ± 18.22	140.74 ± 19.31	142.42 ± 19.19	< 0.001
DBP, mmHg	79.33 ± 10.72	81.03 ± 10.59	82.03 ± 10.38	83.48 ± 10.50	< 0.001
ALT, U/L	18.13 ± 10.68	18.63 ± 10.94	19.74 ± 14.19	22.84 ± 14.07	< 0.001
AST, U/L	22.32 ± 9.86	22.01 ± 8.94	22.01 ± 11.65	23.61 ± 10.78	< 0.001
BUN, mmol/L	5.85 ± 1.54	5.63 ± 1.53	5.55 ± 1.44	5.44 ± 1.43	< 0.001
Scr, *μ*mol/L	72.68 ± 27.09	72.67 ± 21.04	72.52 ± 18.17	72.14 ± 18.32	0.85
SUA, mg/dL	300.97 ± 77.43	312.63 ± 79.52	333.21 ± 82.56	353.09 ± 85.78	< 0.001
eGFR, mL/min/1.73 m^2^	86.16 ± 12.07	84.66 ± 12.36	83.80 ± 14.04	83.45 ± 13.04	< 0.001
FPG, mmol/L	5.32 ± 0.63	5.42 ± 0.64	5.48 ± 0.66	5.59 ± 0.67	< 0.001
TC, mg/dL	171.77 ± 32.39	182.23 ± 33.47	189.66 ± 34.88	195.06 ± 37.81	< 0.001
TG, mg/dL	72.22 ± 13.56	103.87 ± 13.13	141.52 ± 18.96	266.30 ± 154.71	< 0.001
Hypertension	791 (41.76%)	877 (46.30%)	1001 (52.82%)	1058 (55.83%)	< 0.001
Cardiovascular disease	71 (3.75%)	80 (4.22%)	89 (4.70%)	78 (4.12%)	0.54
WTI	7.93 ± 0.20	8.37 ± 0.10	8.71 ± 0.11	9.29 ± 0.36	< 0.001
T2D incidence	103 (5.44%)	152 (8.03%)	209 (11.03%)	294 (15.51%)	< 0.001

*Note:* Continuous variables are shown as mean ± SD, and categorical variables are presented as *n* (%) numbers.

Abbreviations: ALT, alanine transaminase; AST, aspartate transaminase; BMI, body mass index; BUN, blood urea nitrogen; DBP, diastolic blood pressure; eGFR, estimated glomerular filtration rate; FPG, fasting plasma glucose; SBP, systolic blood pressure; Scr, serum creatinine; SUA, serum uric acid; TC, total cholesterol; TG, triglyceride; WC, waist circumference; WTI, waist–triglyceride index.

**Table 3:** Multivariate Cox regression models of the incidence of T2D.

**Variable**	**Univariate analysis**		**Multivariate analysis**	
	**HR (95% CI)**	**p** ** value**	**HR (95% CI)**	**p** ** value**
Age	1.05 (1.04, 1.07)	< 0.0001	1.019 (1.003, 1.034)	0.018
BMI	1.11 (1.09, 1.13)	< 0.0001	1.052 (1.028, 1.077)	< 0.001
FPG	3.86 (3.43, 4.33)	< 0.0001	3.257 (2.884, 3.678)	< 0.001
ALT	1.01 (1.01, 1.01)	< 0.0001	1.013 (1.005, 1.021)	0.001
AST	1.01 (1.00, 1.01)	< 0.0001	0.995 (0.985, 1.004)	0.272
BUN	1.08 (1.03, 1.13)	0.0004	1.047 (1.001, 1.096)	0.047
Scr	1.00 (1.00, 1.00)	0.5186		
SUA	1.00 (1.00, 1.00)	< 0.0001	1.000 (0.999, 1.001)	0.721
eGFR	0.99 (0.98, 0.99)	< 0.0001	1.000 (0.994, 1.007)	0.920
TC	1.00 (1.00, 1.00)	0.3716		
LDL-c	1.00 (0.99, 1.00)	0.0039	0.995 (0.993, 0.998)	< 0.0001
HDL-c	0.98 (0.97, 0.98)	< 0.0001	0.990 (0.984, 0.997)	0.003
SBP	1.01 (1.01, 1.01)	< 0.0001	0.994 (0.988, 1.000)	0.040
DBP	1.01 (1.00, 1.01)	0.0684		
Female	1.32 (1.14, 1.53)	0.0002	1.339 (1.138,1.576)	< 0.001
Smoking				
Never	1			
Former	0.66 (0.41, 1.05)	0.0799		
Current	0.92 (0.77, 1.10)	0.3852		
Drinking				
None	1			
Light	0.70 (0.51, 0.98)	0.0352		
Moderate	0.66 (0.37, 1.21)	0.1791		
Heavy	0.73 (0.57, 0.93)	0.0112		
Hypertension	1.58 (1.37, 1.83)	< 0.0001	1.292 (1.036, 1.611)	0.023
Cardiovascular disease	2.94 (2.34, 3.70)	< 0.0001	2.221 (1.760, 2.802)	< 0.0001
WTI				
Q1	1			0.014
Q2	1.52 (1.18, 1.95)	0.001	1.230 (0.950, 1.592)	0.116
Q3	2.15 (1.70, 2.72)	< 0.0001	1.427 (1.098, 1.855)	0.008
Q4	3.06 (2.44, 3.83)	< 0.0001	1.554 (1.180, 2.048)	0.002

Abbreviations: ALT, alanine transaminase; AST, aspartate transaminase; BMI, body mass index; BUN, blood urea nitrogen; eGFR, estimated glomerular filtration rate; FPG, fasting plasma glucose; HDL-c, high-density lipoprotein cholesterol; LDL-c, low-density lipoprotein cholesterol; Scr, serum creatinine; SUA, serum uric acid; TC, total cholesterol; WTI, waist circumference–triglyceride index.

**Table 4:** The association between WTI and the incidence of T2D during follow-up.

**Type 2 diabetes, HR (95% CI), ** **p** ** value**			
	**Model 1**	**Model 2**	**Model 3**
WTI	2.01 (1.79, 2.25), < 0.001	1.97 (1.76, 2.21), < 0.001	1.57 (1.37, 1.80), < 0.001
WTI quartile			
Q1	Reference	Reference	Reference
Q2	1.52 (1.18, 1.95), 0.001	1.48 (1.15, 1.90), 0.002	1.32 (1.02, 1.70), 0.034
Q3	2.15 (1.70, 2.72), < 0.001	2.07 (1.63, 2.62), < 0.001	1.61 (1.25, 2.07), < 0.001
Q4	3.06 (2.44, 3.83), < 0.001	2.95 (2.35, 3.70), < 0.001	2.02 (1.57, 2.60), < 0.001
*p* for trend	< 0.001	< 0.001	< 0.001

*Note:* Model 1: no covariates were adjusted. Model 2: adjusted for age and gender. Model 3: adjusted for age, gender, smoking status, drinking status, hypertension, cardiovascular disease, BMI, SBP, DBP, FPG, ALT, AST, BUN, Scr, SUA, eGFR, and TC.

Abbreviations: 95% CI, 95% confidence interval; HR, hazard ratio.

## Data Availability

Data was collected from the Kunshan elderly cohort. Data are available upon request from the corresponding authors.
